# Pyrene‐Based “Turn‐Off” Probe with Broad Detection Range for Cu^2+^, Pb^2+^ and Hg^2+^ Ions

**DOI:** 10.1002/chem.202100594

**Published:** 2021-05-10

**Authors:** Viktor Merz, Julia Merz, Maximilian Kirchner, Julian Lenhart, Todd B. Marder, Anke Krueger

**Affiliations:** ^1^ Institute for Organic Chemistry Julius-Maximilians University Würzburg Am Hubland 97074 Würzburg Germany; ^2^ Institute for Inorganic Chemistry Julius-Maximilians University Würzburg, Würzburg Am Hubland 97074 Würzburg Germany; ^3^ Wilhelm Conrad Röntgen Center for Complex Materials Research (RCCM) Julius-Maximilians University Würzburg, Würzburg Am Hubland 97074 Würzburg Germany

**Keywords:** Fluorescence spectroscopy, heavy metals, luminescence, probes, pyrene

## Abstract

Detection of metals in different environments with high selectivity and specificity is one of the prerequisites of the fight against environmental pollution with these elements. Pyrenes are well suited for the fluorescence sensing in different media. The applied sensing principle typically relies on the formation of intra‐ and intermolecular excimers, which is however limiting the sensitivity range due to masking of e. g. quenching effects by the excimer emission. Herein we report a highly selective, structurally rigid chemical sensor based on the monomer fluorescence of pyrene moieties bearing triazole groups. This sensor can quantitatively detect Cu^2+^, Pb^2+^ and Hg^2+^ in organic solvents over a broad concentrations range, even in the presence of ubiquitous ions such as Na^+^, K^+^, Ca^2+^ and Mg^2+^. The strongly emissive sensor's fluorescence with a long lifetime of 165 ns is quenched by a 1 : 1 complex formation upon addition of metal ions in acetonitrile. Upon addition of a tenfold excess of the metal ion to the sensor, agglomerates with a diameter of about 3 nm are formed. Due to complex interactions in the system, conventional linear correlations are not observed for all concentrations. Therefore, a critical comparison between the conventional Job plot interpretation, the method of Benesi‐Hildebrand, and a non‐linear fit is presented. The reported system enables the specific and robust sensing of medically and environmentally relevant ions in the health‐relevant nM range and could be used e. g. for the monitoring of the respective ions in waste streams.

## Graphical abstract

**An efficient pyrene‐based probe** using fluorescence quenching to detect mercury, lead and copper in organic solvents with high selectivity and specificity was synthetized. The rigid tris‐pyrene chromophore architecture ensures a very broad dynamic range of the sensor's response. Spectroscopic investigation elucidated the stoichiometry and mode of action and revealed a unique aggregation behavior at high analyte concentrations.

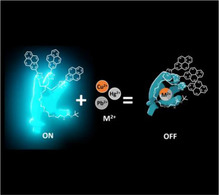

## Introduction

The use of heavy metals in industry, agriculture, household and technology is one of the important sources of environmental pollution and detrimental effects on plants and the health of animals and humans.[^1^, ^2^, ^3^, ^4^, ^5^, ^6^, ^7^] Metals such as copper, lead and mercury are among the most common in waste water.^[8]^ Copper is very common, not only in industry, but also in the home (copper pipes, etc.) and as a pesticide that is released directly into the environment. It causes diseases such as Wilson's disease and the so‐called “vineyard sprayer‘s lung”.[^9^, ^10^, ^11^] Sources of lead pollution in the environment are manifold. The majority originate from the melting and processing of ores, but industrial and car exhaust gases, lead batteries, additives in paints and varnishes, and petrol additives that were used until the 2000’s are also sources.[^6b^, ^12^] Consequential damage from prolonged exposure to these pollutants include reduced intelligence, delayed or impaired neurobehavioral development, reduced hearing acuity, speech impairment, and growth disorders, even at very low exposure.^[6]^ Mercury had its peak in industrial demand when numerous measuring instruments, such as thermometers, barometers, etc., as well as lighting tubes were manufactured using this metal. When it is released into the environment, mercury is broken down by microorganisms into toxic methyl mercury compounds and it accumulates in the food chain which causes the so‐called Minamata disease.[^6^, ^7^, ^13^, ^14^]

Once heavy metals are ingested, their ability to be reduced or oxidized enables them to bind to biomolecules in various oxidation states and then alter or even block important functions of vital metal complexes, thereby circumventing control mechanisms of the organism and leading to toxic effects.[^5^, ^7^, ^15^, ^16^]

In order to prevent the release of heavy metals into the environment as early as possible, control mechanisms must be implemented whenever these metals are used.[^6^, ^17^, ^18^] As heavy metals in industry are not only applied in an aqueous environment, whereas it is crucial to have water soluble sensors, there is a need for sensors applicable in different solvents. It is therefore important to manufacture specific sensors for applications in different media. In order to address the diverse range of uses of heavy metals, and to detect and eliminate them quickly in the event of uncontrolled release into the environment, tailored sensors are required.

In this work, we present a pyrene‐ and tetraethylene glycol (TEG)‐based molecular sensor for selectively detecting Cu(II), Pb(II) and Hg(II) in health‐relevant concentrations via quenching of the monomer fluorescence of pyrene in organic solvents.

## Results and Discussion

The reported sensor combines a previously reported glycol precursor^[19]^ with a rigid arrangement of pyrene moieties. Due to its versatile and extensively investigated properties, pyrene is well suited as a chemical sensor.[^20^, ^21^, ^22^, ^23^, ^24^, ^25^, ^26^, ^27^, ^28^, ^29^] Pyrene derivatives functionalized at the 2‐position via a one‐step iridium‐catalyzed reaction with B_2_pin_2_ offer significantly longer fluorescence lifetimes in comparison to pyrene derivatives that are functionalized at the 1‐position.[^30^, ^31^] As a chemosensor, in most cases, reports on the analyte in its excited state, the response is enhanced for sensors with longer excited state lifetimes, leading to improved sensitivity.^[32]^ However, until now, only a few chemosensors based on pyrenes functionalized at their 2‐position have been reported.[^27^, ^28^, ^33^, ^34^] These sensors were designed to enter into targeted interactions with metals through functional groups or crown ethers on the pyrene. In our work, we combine pyrene functionalized at its 2‐position with a tailored glycol chain, which has two decisive advantages. First, the molecule is soluble and can be purified via solution‐phase methods such as chromatography and applied in a variety of solvents. Second, TEG provides crown ether‐like properties which improve coordination to metal ions.^[35]^ Therefore, the sensor was constructed using a TEG chain carrying a branching moiety that bears three binding sites for pyrenyl groups bound at their 2‐position.

### Synthesis and characterization of a rigid tris‐pyrenyl sensor

**Scheme 1:**
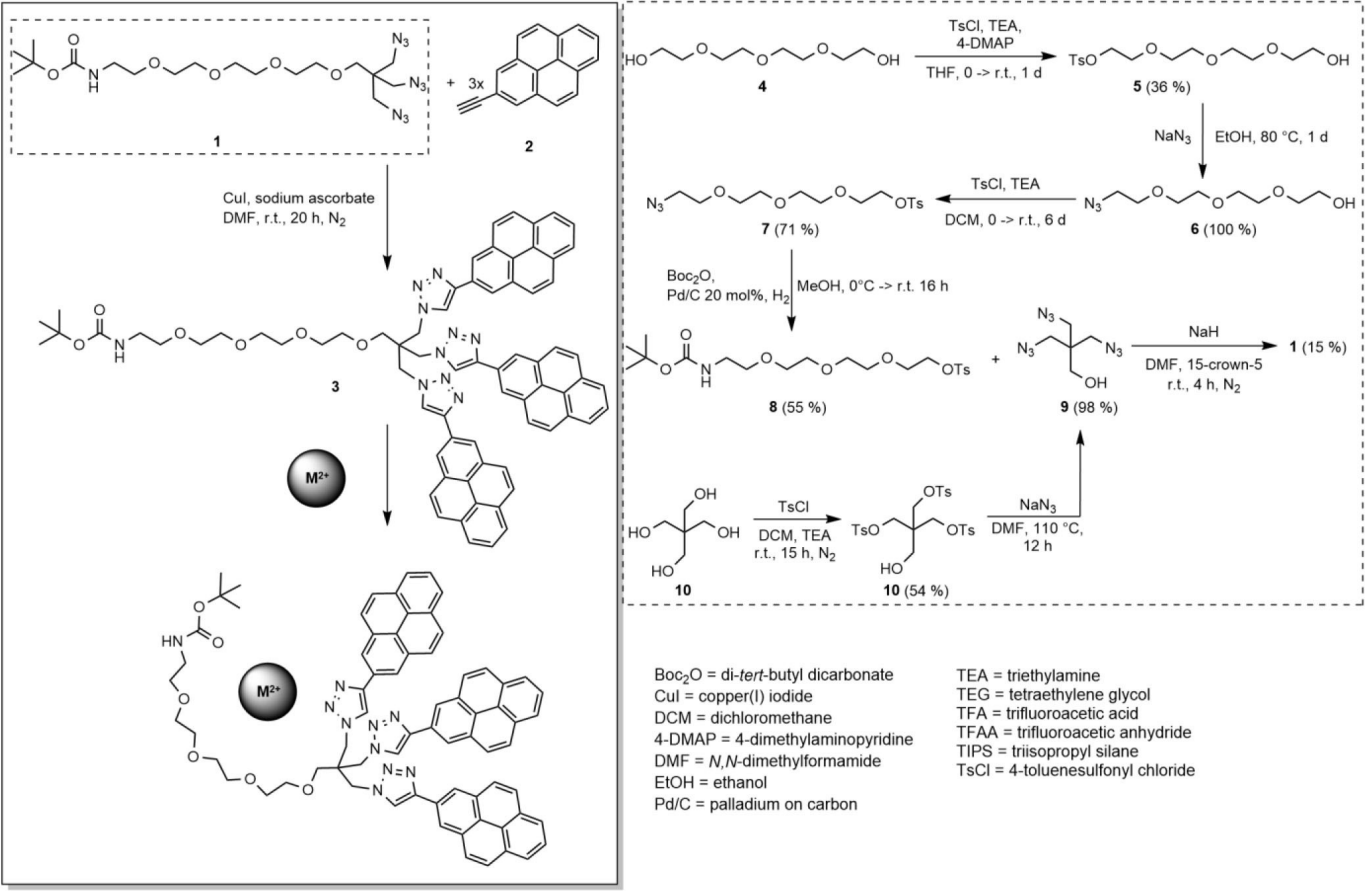
Synthesis of the glycol chain (dashed rectangle) and of probe **3** using click chemistry and its mode of action when complexing a divalent M^2+^ cation in acetonitrile.

**Figure 1:**
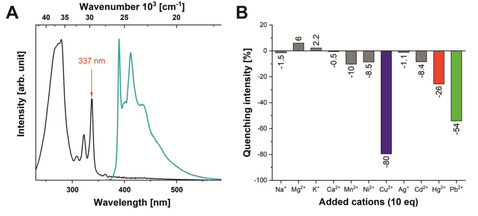
A) Normalized absorption and emission (excited at 337 nm) spectra of probe **3** in acetonitrile (concentration: 3.17×10^−6^ mol L^−1^) and B) fluorescence responses of **3** (concentration: 1.02×10^−6^ mol L^−1^) to a selection of monovalent and divalent metal ions (used in the form of perchlorate salts) at 10 eq excess (see Supporting Information for a complete set of tested cations).

The TEG chain **1** is used as the scaffold for the attachment of three pyrenes via the formation of triazole linkers in a click reaction of **1** with three molecules of 2‐ethynylpyrene **2** (Scheme 1). The resulting tris‐pyrenyl conjugate not only possesses the ability to complex metal ions, but the opposite terminus is designed to allow for subsequent immobilization of the sensor using the amino function for coupling to, e. g., nanoparticles, chip surfaces or biomolecules. A very important feature for the functionality of this probe is the reduced spacing and the ensuing low steric flexibility at the focal point of the molecule's branching unit, preventing **3** from forming *intra*molecular dimers of the pyrene head groups. The interaction between two molecule head groups to form an *inter*molecular dimer is also suppressed by the limited flexibility of the branching unit. These structural features allowed us to apply this sensor in a very broad concentration range (see below). Previously, Seela and Ingale reported a pyrene‐based sensor for the detection of Zn^2+^, which shows very efficient excimer formation due to the flexibility of the different pyrene moieties.^[36]^ However, due to its structural design, our probe does not show excimer emission that could mask possible signals in the longer‐wavelength range in solvents such as MeOH, MeCN, CH_2_Cl_2_ and toluene from low to even very high concentrations (Supporting Information Figure S4 and Figure 1). However, for applications such as fluorescent ratiometric detection pyrene's excimer emission can be of interest.^[36]^

Pyrene excimer formation is usually observed in the wavelength range between 400–600 nm, with a maximum at about 500 nm, and is used in many applications for sensing due to its fluorescence signal strength and sensitivity.[^25^, ^26^, ^27^, ^37^, ^38^, ^39^, ^40^, ^41^] However, when sensing depends on longer lifetimes of the excited state, a chemical sensor relying on monomer fluorescence is superior to a sensor based on excimer formation. Depending on the system, the lifetime of an excimer of pyrene is typically about one order of magnitude shorter than that of the excited monomer state.^[42]^

The absorption and emission spectra of **3** are shown in Figure 1. The spectral features of pyrenes are clearly visible.

The absorption spectrum of unsubstituted pyrene consists of four bands,^[43]^ one forbidden band (S_1_←S_0_) at 372 nm (ϵ=510 mol^−1^ cm^−1^ l) with vibrational fine structure, two bands (S_2_←S_0_ and S_3_←S_0_) at 334 and 272 nm, and a strongly allowed band (S_4_ ←S_0_) at 243 nm (ϵ=88000 mol^−1^ cm^−1^ l).[^30^, ^43^] In this work, the absorption of the probe is very similar to that of unsubstituted pyrene due to the functionalization at 2‐position of pyrene, which is situated on nodal planes in both the HOMO and LUMO, previously studied in detail by Marder and co‐workers.[^30^, ^44^] Thus, the S_1_←S_0_ transition is still forbidden (372 nm) and the S_2_←S_0_ transition is slightly bathochromically shifted with a maximum at 337 nm.

In a screening experiment, the effect of a large number of cations on the fluorescence of **3** was investigated (Figure S12). The metal ions leading to significant changes and the most typical background ions were again measured as perchlorate salts, as these offer good solubility in acetonitrile and do not absorb light in the region of the sensor's fluorescence, avoiding reabsorption of emitted light. Strong fluorescence quenching was observed in the presence of Cu^2+^, Pb^2+^ and Hg^2+^ while the emission wavelength was not influenced. Moreover, no quenching effects were observed upon addition of Ca^2+^, Mg^2+^, Na^+^ and K^+^, which makes the sensor interesting for applications in which solvents come into contact with ions that are typically present in aqueous environments.

### Fluorescence titrations

**Figure 2:**
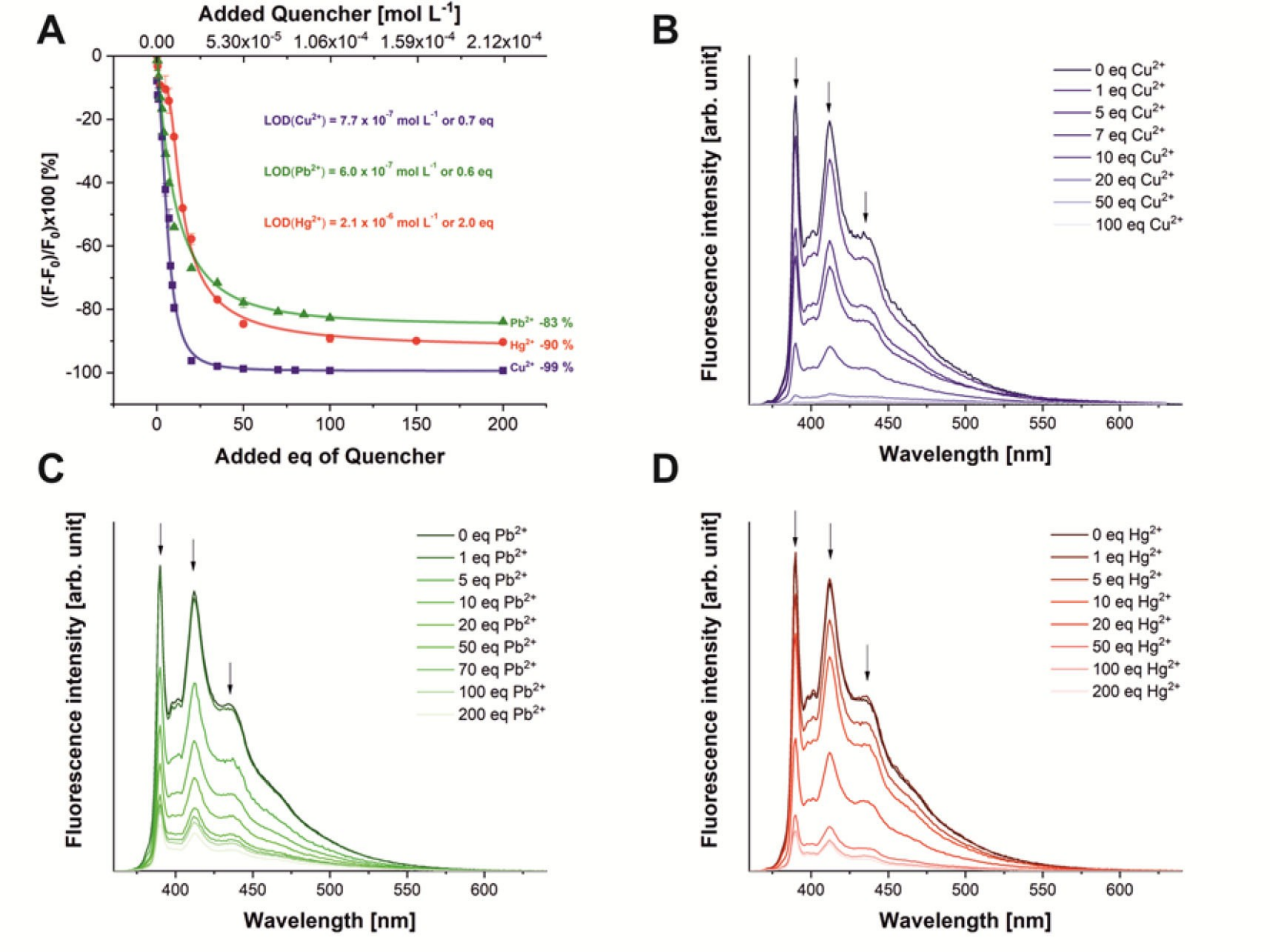
A) Fluorescence titration spectra of sensor **3** at 1.02×10^−6^ mol L^−1^ with the quencher ions Cu^2+^(blue squares), Pb^2+^ (green triangles) and Hg^2+^ (red dots), error bars are given for all data points (see Figure S13 for magnification); individual spectra for the titration of B) copper, C) lead, D) mercury. All cations were used in the form of their perchlorate salts.

Titrations of the three analyte cations (Figure 2) show strong, concentration‐dependent quenching until a plateau of −83 (Pb^2+^), −90 (Hg^2+^) and −99 (Cu^2+^) %, of their initial luminescence intensity is reached.

The detection limit (LOD) describes the lower limit at which the sensor can reliably measure the ions. According to the definition, this is achieved if the measurement signal is at least 3.3 times higher than the standard deviation of the measurement.^[45]^ Here, the LOD was determined for a sensor concentration of 1.02×10^−6^ mol L^−1^ leading to an optical density of 0.1. The strongest effect on the fluorescence of **3** (at a concentration of 1.02×10^−6^ mol L^−1^, 1 eq) is observed upon addition of Cu^2+^ translating to an LOD of 7.7×10^−7^ mol L^−1^ (0.7 eq), Pb^2+^ can be measured at concentrations from 6×10^−7^ mol L^−1^ (0.6 eq) and Hg^2+^ from 2.1×10^−6^ mol L^−1^ (2 eq) equivalents with confidence, which allows the sensing of all these ions in the health‐relevant concentration range. However, as can be seen in the titrations carried out for the determination of the complex’ stoichiometry (see below), the system delivers precise values also in the single‐digit nM range, when the concentration of sensor **3** is reduced to 1.06×10^−10^ mol L^−1^.

For all sensor concentrations, quantitative statements can be made in a range of up to 50 eq. of the quencher ions, thus defining the upper limit of quantification (ULOQ). As can be seen in Figure 2A, the linear part of the dynamic range of the sensor covers the ratio of 0 to 10 eq. of quencher (see below for a detailed discussion of the non‐linear behavior). Therefore, the ULOQ has to be determined by visual inspection of the titration curves, leading to ULOQ values of 20 eq. for Cu^2+^ (2.1×10^−5^ mol L^−1^) and for Pb^2+^ and Hg^2+^ of 50 eq (5.3×10^−5^ mol L^−1^) for a sensor concentration of 1.02×10^−6^ mol L^−1^.

As the spectroscopic characteristics of the sensor fluorescence are identical for concentrations from 1.06×10^−10^ mol L^−1^ to 3.17×10^−6^ mol L^−1^ due to the absence of excimer formation, the concentration of **3** can be adjusted to the amount of quencher ions in the analyte solution. The saturation plateau for the three ions Pb^2+^, Hg^2+^ and Cu^2+^ is reached at −83 %, −90 % and −99 % of the initial fluorescence intensity, respectively.

The observed sensor properties are related to its mode of action. The system is based on dynamic quenching, which is the interaction of a quencher with a molecule in an excited state. Therefore, the sensitivity of the sensors strongly depends on the lifetime of the excited state from which the observed emission originates.^[46]^ Thus, the observed very long fluorescence lifetime of **3** (165 nm) is beneficial for an efficient sensing process if dynamic quenching is the mode of action.

### Fluorescence lifetime measurements

**Figure 3:**
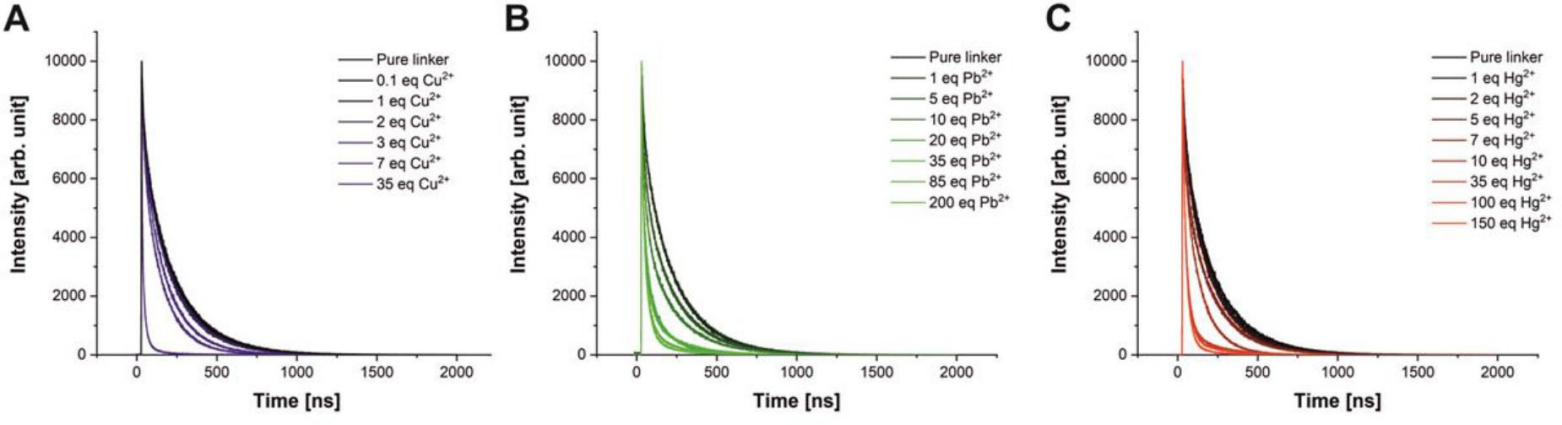
Lifetime titration spectra of sensor **3** upon addition of A) Cu^2+^, B) Pb^2+^ and C) Hg^2+^.

As shown in Figure 3, the fluorescence lifetime of sensor 3 is strongly dependent on the ion concentration. This can be used to confirm the type of fluorescence quenching process.

If dynamic quenching is involved, the decrease in lifetime is proportional to the concentration of the quencher.^[46]^ After reaching the ULOQ, the lifetimes decrease for **3** to 5 ns quenched by Cu^2+^, to 47 ns by Pb^2+^ and to 36 ns by Hg^2+^. Additionally, the characteristics of the dynamic quenching process, like the mode and rate of interaction between host and quencher, need to be investigated.

### Job plots

**Figure 4:**
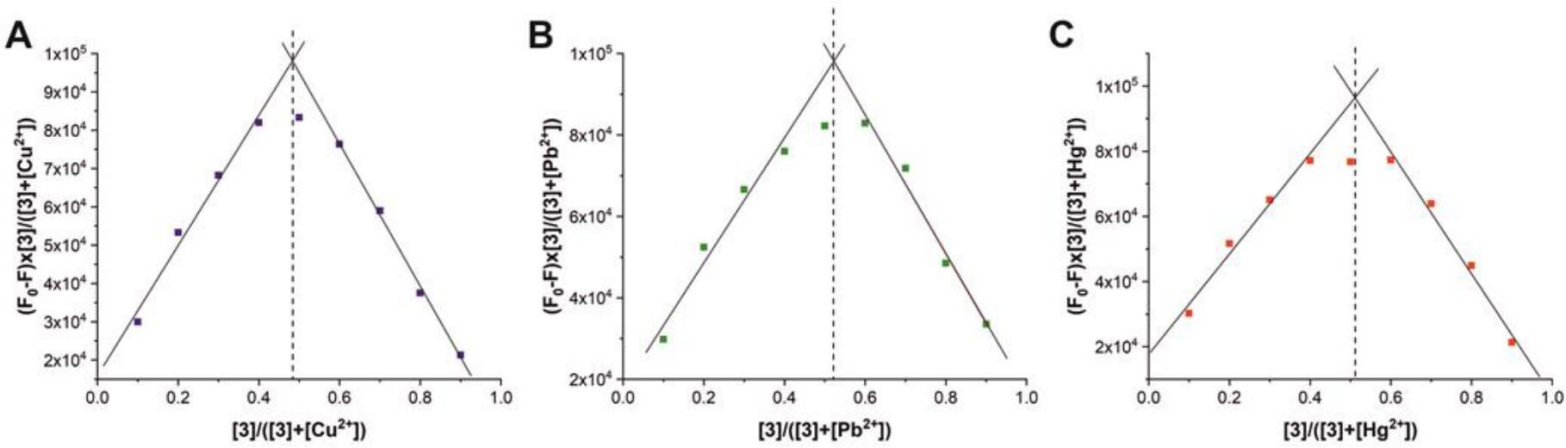
Job plot for each metal ion A) Cu^2+^, B) Pb^2+^ and C) Hg^2+^ for a titration with a constant total host and guest concentration of 1.06×10^−9^ mol L^−1^ in the concentration ratio 9 : 1 to 1 : 9 in integer steps.

A commonly used method to determine the interaction stoichiometry of the host sensor with a guest ion, is the Job plot, which is shown in Figure 4.[^47^, ^48^] However, this plot cannot be used to display systems with stoichiometries other than 1 : 1. In 1 : 2 or 2 : 1 complexes the maximum of the plot does not necessarily show the actual binding stoichiometry. In cases where the quotient of K_1:1_/K_1:2_ (or K_1:1_/K_2:1_) is larger than 1, the Job plot cannot be applied. Here, the maximum at 0.5 indicates a 1 : 1 ratio, which has only one binding constant, thus the application of the Job plot is justified.^[49]^ This result has been confirmed using the free *bindfit* software, where a 1 : 1 stoichiometry gave highly satisfactory results (http://supramolecular.org). The fit for hypothetical 1 : 2 and 2 : 1 complexes resulted in negative values for binding constants and/or unrealistically high error values, while the unconstrained fit for a 1 : 1 system coincided with the results of the linear fits (see Figure S5 and S6).

**Figure 5:**
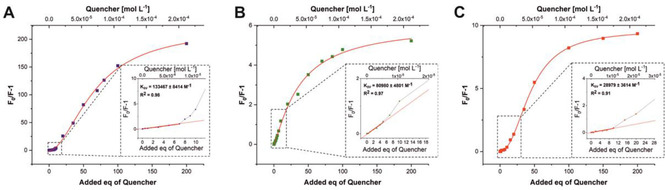
Stern‐Volmer plots of A) Cu^2+^, B) Pb^2+^ and C) Hg^2+^ titrated to a constant host **3** concentration (1.02×10^−6^ mol L^−1^_,_ 1 eq) from 0–200 eq of analyte. The insets show the linear regions at low quencher concentration.

In addition to binding constants, the total concentration of the sensor is also a crucial factor for this type of analysis. The more concentrated the system, the more reliable values can be read from the Job plot.^[47]^ However, for fluorescence measurements, the concentration range is usually determined by two factors: the detection window is limited on one side by the fluorescence detection limit and on the other side by the prevention of self‐quenching of the fluorophores. In the case of pyrene derivatives, excimer formation is also observed in concentrated solutions.^[29]^ By design, the system presented here has no possibilities to form an excimer, as already discussed. However, an unforeseen aspect of the molecular structure has been elucidated using Stern‐Volmer plots (Figure 5).

### Stern‐Volmer plots

An important prerequisite for the validity of the Stern‐Volmer equation is the equal accessibility of all molecules of the fluorophore by the quencher, i. e. the same Stern‐Volmer constant must apply to all molecules of the fluorophore.^[50]^ If this precondition is not applicable due to the presence of differently accessible fluorophores, the Stern‐Volmer equation cannot be used in its usual form. In the system presented here, all titrations can be linearly fit in the Stern‐Volmer plot for the range of 0–7 eq (0–7×10^−6^ mol L^−1^) of quencher.

The quenching constant, the Stern‐Volmer constant (*K*_SV_), can be read from the slope of the straight line. The slope for Cu^2+^ is 1.3×10^5^ M^−1^, for Pb^2+^8.1×10^4^ M^−1^ and for Hg^2+^2.9×10^4^ M^−1^. For concentrations above 7 eq, however, the slope of the curve increases. Values for F_0_/F‐1 in the Stern‐Volmer plot deviating from linearity to higher values indicate the presence of processes that lead to a more efficient quenching of the fluorescence at higher quencher concentrations.

**Figure 6:**
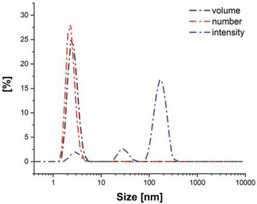
DLS size measurement (diameter) in acetonitrile of probe **3** (5×10^−6^ mol L^−1^_,_ 1 eq) in the presence of 200 eq of Cu^2+^. To identify the different size classes of aggregates, different presentation modes of the scattering signal are shown. Note: Large objects are overrepresented in the intensity plot due to their higher scattering cross section (scaling with r^6^) whereas in the number plot, larger objects are not visible due to their low concentration.

These results suggest that a further quenching effect comes into play at higher ratios of guest ions to host sensor **3**. As the formation of excimers can be excluded from concentration‐dependent measurement of **3**, we hypothesized that larger aggregates are formed. In fact, with the help of dynamic light scattering (DLS) experiments (Figure 6), metal ion‐induced aggregate formation was confirmed that is not observed for solutions of sensor molecule **3** in the absence of metal ions. The formation of flocculates upon the generation of an iron complex of a pyrene sensor that led to fluorescence quenching has been reported.^[51]^

The aggregation process was found to be reversible as shown by the disappearance of the agglomerates upon addition of an equally concentrated solution of **3** until the concentration of Cu^2+^ is reduced to 60 eq, and their slow reformation after about 30 min as shown by DLS (see Figure S9 for details). As can be deduced from the volume distribution determined by DLS, 90 % of the agglomerates are smaller than 3.5 nm in diameter. The simultaneous presence of larger objects in the size range of 30 and 200 nm is evidenced by the scattering peaks in the intensity distribution. This confirms the suspected complex agglomeration behavior of the sensor molecule. In previous agglomeration studies similar observations have been reported for titrations and the associated Stern‐Volmer plots.[^50^, ^52^, ^53^, ^54^]

### Comparison with Benesi‐Hildebrand plots

Another frequently applied method to characterize the binding between host and analyte is the Benesi‐Hildebrand method, where the binding constant (K_a_) can be determined from the double reciprocal plot of the fluorescence ratio over the quencher concentration (see Figure S10).^[55]^

As for the Stern‐Volmer plot, the Benesi‐Hildebrand plots of **3** exhibits the different interaction regimes at low and high analyte concentrations. For Cu^2+^ and Pb^2+^, the linearity is preserved in the low concentration range, the results for Hg^2+^ are not fully congruent. However, it turns out that in the sensitive area of the sensor (0–10 eq), the linearity is not sufficiently accurate to use the results of the Benesi‐Hildebrand method to determine the binding constants. At higher concentrations (>20 eq), all three systems show linearity. However, the binding constants calculated from these slopes differ greatly from the results obtained with the *bindfit* method for the region of >10 eq (Table 1).

**Table 1:** Quenching constants *K*_SV_ obtained from Stern‐Volmer‐plots and binding constants K_a_ obtained from Benesi‐Hildebrand (BH) plot and the software *bindfit* for low and high quencher concentrations.

Quencher	eq	K_SV_ [M^−1^]	*K*_a(BH)_ [M^−1^]	*K*_a(bindfit)_ [M^−1^]
Cu^2+^	<10	1.3×10^5^	1.26×10^5^	1.54×10^5^
Cu^2+^	>10		1.21×10^6^	3.96×10^4^
Pb^2+^	<10	8.1×10^4^	2.25×10^4^	1.10×10^4^
Pb^2+^	>10		1.55×10^5^	2.18×10^4^
Hg^2+^	<10	2.9×10^4^	2.02×10^5^	9.16×10^5^
Hg^2+^	>10		5.69×10^4^	2.99×10^4^

The results for the respective binding constants, determined via the Benesi‐Hildebrand‐plot and *bindfit*, suggest that the Benesi‐Hildebrand plot fails to adequately describe a quenching method that consists of dynamic quenching and agglomeration, which has also been discussed previously.^[48]^

### NMR titrations

In order to obtain more information about the binding constants in addition to the fluorescence titration experiments, we performed NMR titration experiments in DMSO‐d_6_ to investigate the ^1^H‐NMR shifts upon addition of the three quenchers. The results are presented in the Supporting Information (Figure S11). They suggest that the interaction of the metal ions is not limited to the pyrene moiety and the triazole unit, but also involves the TEG chain, as indicated by the concomitant strong ^1^H NMR shift. The NMR experiments also confirm an identical binding mode for all three analyte ions (see details in Supporting Information). In the concentration range that was observable by NMR spectroscopy (0–50 eq), no saturation effect was found, thus confirming the assumed dynamic interaction between the ions and the sensor.

## Conclusion

A highly specific, pyrene‐based turn‐off probe for the detection and quantification of the environmentally important metals Cu^2+^, Pb^2+^ and Hg^2+^ was developed. The substitution of pyrene at its 2‐position instead of the usual 1‐position led to a rare probe with long fluorescence lifetimes enabling investigation of the dynamic quenching mechanism. Furthermore, the rigidity of the sensor architecture prevents the otherwise prominent excimer formation of the pyrenes and thereby allows the study of the monomer emission. A 1 : 1 complex stoichiometry was confirmed using *bindfit* and Job plots, whereas the Stern‐Volmer plots revealed a complex set of interactions involving not only the formation of the stoichiometric complex, but also the reversible, metal‐induced formation of nanosized aggregates at higher quencher concentrations. Binding constants in the range of 1.10×10^4^–9.16×10^5^ m ^−1^ allow for the sensitive quantification of the ions over a broad concentration range in organic solvents. In the future, the system could be immobilized on a solid support to prevent aggregate formation and, moreover, make the system applicable in aqueous environments. Such applications are already underway in our laboratory.

## Experimental Section

### Synthetic and spectroscopic procedures

**General methods**: Azide **1** and alkyne **2** were prepared according to literature procedures.[^19^, ^44^] All other starting materials were purchased from commercial sources and used as received. The solvents were freshly distilled and dried using standard procedures. Nuclear magnetic resonance spectra (^1^H and ^13^C) were measured with a Bruker AVANCE 400 FT‐NMR spectrometer at 27 °C. For the calibration of spectra, the chemical shift of the deuterated solvents was used as internal reference. HRMS were recorded using a Thermo Scientific Exactive PlusOrbitrap MS system with either an Atmospheric Sample Analysis Probe (ASAP) or by Electrospray Ionization. The characterization by Fourier‐transform infrared spectroscopy (FTIR) was carried out using a Jasco FT/IR‐430 spectrometer fitted with an attenuated total reflection (ATR) MIRacle unit from PIKE Technologies. The particle size was measured using a Malvern Zetasizer Nanoseries Nano‐ZS (dynamic light scattering, backscattering mode). The size distribution is given as volume, number and intensity distribution and was obtained using the Marquardt method. Degassing of solutions was performed by sonication using a Bandelin Sonorex Digitec Typ DT52 sonicator bath (max. 80 W, 35 kHz). All photophysical measurements were carried out under an argon atmosphere by preparing the samples in an argon‐filled glovebox. The solution state measurements were performed in standard quartz cuvettes (1 cm×1 cm cross section). The UV/Vis absorption spectra were measured using an Agilent 1100 diode array UV/Vis spectrophotometer. Excitation, emission, lifetime and quantum yield measurements were recorded using an Edinburgh Instruments FLSP920 spectrometer equipped with a 450 W Xenon arc lamp, double monochromators for the excitation and emission pathways, and a red‐sensitive photomultiplier (PMT−R928P) as the detector. The measurements were performed in right‐angle (90°) geometry mode and all spectra were fully corrected for the spectral response of the instrument.

Fluorescence quantum yields of the samples were measured using a calibrated integrating sphere (150 mm inner diameter) from Edinburgh Instruments attached to the FLSP920 spectrometer described above. For solution‐state measurements, the longest wavelength absorption maximum of the compound in the respective solvent was chosen for the excitation. In order to exclude self‐absorption, the emission spectra were measured with dilute samples (approx. 0.1 optical density at the excitation wavelength).

Fluorescence lifetime measurements were conducted using the time‐correlated single‐photon counting method (TCSPC) on the FLSP920 spectrometer equipped with a high‐speed photomultiplier tube positioned after a single emission monochromator. Measurements were made in right‐angle (90°) geometry mode, and the emission was collected through a polarizer set to the magic angle. Solutions were excited with a 315 nm (pulse width 932.5 ps) pulsed diode laser at repetition rates of 1–5 MHz and were recorded at the emission maxima. Decays were recorded to 10,000 counts in the peak channel with a record length of at least 4,000 channels. The band‐pass of the monochromator was adjusted to give a signal count rate of <20 kHz. Iterative reconvolution of the instrument response function (IRF) with one decay function and nonlinear least‐squares analysis were used to analyze the data. The quality of all decay fits was judged to be satisfactory, based on the calculated values of the reduced χ^2^ and Durbin‐Watson parameters and visual inspection of the weighted and autocorrelated residuals.

The titration experiments for the Job plot were carried out in a 0 : 10–10:0 ratio (c/c), the total concentration of all components always being 1.06×10^−9^ mol L^−1^. For all other titrations, the concentration of the host was kept constant at 1.02×10^−6^ mol L^−1^ and 0–200 eq of the quencher was added. All titrations were carried out at least in triplicate to obtain a standard deviation.

**Synthesis of 3**: Under an N_2_ atmosphere, azide **1** (0.80 g, 1.6 mmol, 1.0 eq) and alkyne **2** (1.50 g, 6.6 mmol, 4.1 eq) were dissolved in DMF (20 mL) and degassed under a stream of nitrogen in an ultrasonic bath for 15 min. Sodium ascorbate (1.31 g, 6.6 mmol, 4.1 eq) and CuI (0.42 g, 1.6 mmol, 1.0 eq) were then added and the mixture was stirred at room temperature for 20 h. The solvent was removed, and the residue was dissolved in CH_2_Cl_2_ and water. The organic phase was washed with brine and the combined aqueous solutions extracted with CH_2_Cl_2_. The combined organic phases were dried over magnesium sulphate and concentrated under reduced pressure. The crude product was purified by column chromatography (eluent CyH/EtOAc, 1 : 1→ 0 : 1, v/v). After evaporation of the solvent, product **3** was obtained as a yellowish solid. **Yield**: 0.6 g (0.51 mmol, 32 %). M.p.: 180–220 °C. **R**_***f***_ (cyclohexane/EtOAc, 1 : 1): 0.05. ^**1**^ **H NMR** (400 MHz, CDCl_3_): δ=8.94 (s, 3H, H‐12), 8.73 (s, 6H, H‐7), 8.16 (d, ^3^ *J*_2,1_=7.6 Hz, 6H, H‐2), 8.13–8.03 (m, 12H, H‐4+5), 7.99 (dd, ^3^ *J*_1,2_=7.6 Hz, 3H, H‐1), 4.81 (br, 1H, H‐24), 4.69 (s, 6H, H‐13), 3.90–3.81 (m, 4H, H‐18/19/20/21), 3.79–3.74 (m, 2H, H‐18/19/20/21), 3.74–3.64 (m, 2H, H‐18/19/20/21), 3.42–3.33 (m, 2H, H‐16), 3.32–3.27 (m, 2H, H‐17), 3.27 3.19 (m, 2H, H‐22), 3.18–3.01 (m, 4H, H‐15+23), 1.36 (s, 9H, H‐27) ppm. ^**13**^ **C NMR** (100 MHz, CDCl_3_): δ=156.0 (C_q_, C‐25), 148.2 (C_q_, C‐11), 131.8 (C_q_, C‐8), 131.2 (C_q_, C‐6), 128.1 (CH, C‐4), 127.9 (C_q_, C‐3), 127.5 (CH, C‐5), 126.2 (CH, C‐1), 125.4 (CH, C‐2), 124.7 (C_q_, C 9/10), 124.6 (C_q_, C 9/10), 124.2 (CH, C‐12), 122.1 (CH, C 7), 79.3 (C_q_, C 26), 77.4 (CH, C‐16), 70.8 (CH_2_, C 17–22), 70.7 (CH_2_, C 17–22), 70.6 (CH_2_, C 17–22), 70.5 (CH_2_, C‐17–22), 70.1 (CH_2_, C 17–22), 70.0 (CH_2_, C 17–22), 68.3 (CH_2_, C‐15), 49.7 (CH_2_, C‐13), 46.5 (C_q_, C‐14), 40.2 (CH_2_, C‐23), 28. 5 (CH_3_, C‐27) ppm. **FT‐IR (ATR)**: $\tilde{v}$ =3127 (w), 3039 (w), 2966 (w), 2868 (m), 1704 (vs), 1608 (m), 1504 (s), 1437 (s), 1365 (s), 1275 (m), 1245 (vs), 1170 (vs), 1137 (vs), 1095 (vs), 1040 (vs), 1006 (w), 962 (vw), 879 (vs), 839 (vs), 819 (vs), 759 (m), 727 (s), 706 (vs), 660 (m), 608 (w) cm^−1^. **HRMS** (ESI,+): found: 1164.5000 [M]^+^; calc. for [M]^+^: 1164.5010.

## Conflict of interest

The authors declare no conflict of interest.

## Supporting information

As a service to our authors and readers, this journal provides supporting information supplied by the authors. Such materials are peer reviewed and may be re‐organized for online delivery, but are not copy‐edited or typeset. Technical support issues arising from supporting information (other than missing files) should be addressed to the authors.

Supplementary
