# Supplementary material for: Pyrene‐Based “Turn‐Off” Probe with Broad Detection Range for Cu2+, Pb2+ and Hg2+ Ions
Source: Chemistry. 2021 May 10;27(31):8118–26. doi: 10.1002/chem.202100594 (PMC8251986; doi:10.1002/chem.202100594)
Supplement: Supplementary file 1 — Supplementary [file CHEM-27-8118-s001.pdf]

Chemistry–A European Journal

Supporting Information

Pyrene-Based "Turn-Off" Probe with Broad Detection Range for Cu^{2+}, Pb^{2+} and Hg^{2+} Ions

Viktor Merz, Julia Merz, Maximilian Kirchner, Julian Lenhart, Todd B. Marder, and Anke Krueger*

Table of contents

Synthetic and spectroscopic details of compound 3: p. 2

Snapshots of the data obtained from bindfit: p. 6

DLS measurements: p. 10

Benesi-Hildebrand-Plot p. 11

^{1}H NMR titrations p. 12

Screening experiments using different non-perchlorate salts p. 13

Magnification of Figure 2A: p. 14

Synthesis:

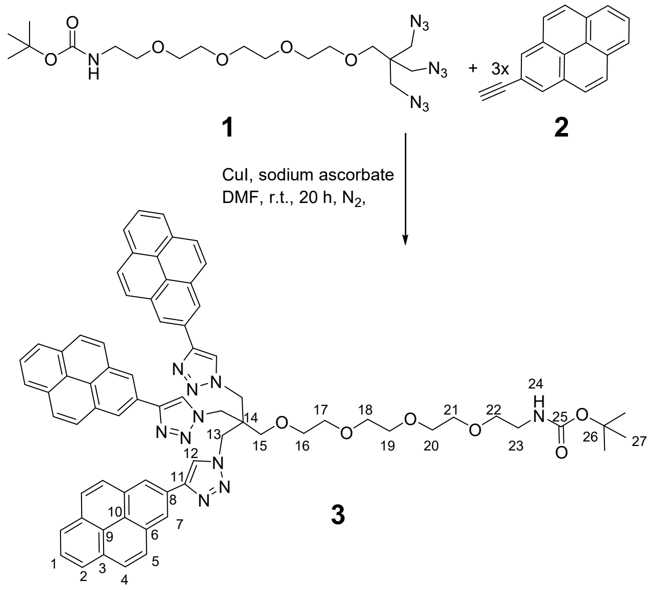

Under an N_2 atmosphere, azide **1** (0.80 g, 1.6 mmol, 1.0 eq) and alkyne **2** (1.50 g, 6.6 mmol, 4.1 eq) were dissolved in DMF (20 mL) and degassed under a stream of nitrogen in an ultrasonic bath for 15 min. Sodium ascorbate (1.31 g, 6.6 mmol, 4.1 eq) and CuI (0.42 g, 1.6 mmol, 1.0 eq) were then added and the mixture was stirred at room temperature for 20 h. The solvent was removed, and the residue was dissolved in CH_2Cl_2 and water. The organic phase was washed with brine and the combined aqueous solutions extracted with CH_2Cl_2 were dried with magnesium sulfate and concentrated under reduced pressure. The crude product was purified by column chromatography (eluent CyH / EtOAc, 1:1 → 0:1, v/v). After evaporation of the solvent, **3** was obtained as a yellowish solid.

Yield: 0.6 g (0.51 mmol, 32 %). M.p.: 180-220 °C. $\mathbf{R_f}$ (cyclohexane/EtOAc, 1:1): 0.05. $\mathbf{^1H}$ **NMR** (400 MHz, $CDCl_3$): δ = 8.94 (s, 3H, H-12), 8.73 (s, 6H, H-7), 8.16 (d, $^3J_{2,1}$ = 7.6 Hz, 6H, H-2), 8.13-8.03 (m, 12H, H-4+5), 7.99 (dd, $^3J_{1,2}$ = 7.6 Hz, 3H, H-1), 4.81 (br, 1H, H-24), 4.69 (s, 6H, H-13), 3.90-3.81 (m, 4H, H-18/19/20/21), 3.79-3.74 (m, 2H, H-18/19/20/21), 3.74-3.64 (m, 2H, H-18/19/20/21), 3.42-3.33 (m, 2H, H-16), 3.32-3.27 (m, 2H, H-17), 3.27 3.19 (m, 2H, H-22), 3.18-3.01 (m, 4H, H-15+23), 1.36 (s, 9H, H-27) ppm. $\mathbf{^{13}C}$ **NMR** (100 MHz, $CDCl_3$): δ = 156.0

(C_q, C-25), 148.2 (C_q, C-11), 131.8 (C_q, C-8), 131.2 (C_q, C-6), 128.1 (CH, C-4), 127.9 (C_q, C-3), 127.5 (CH, C-5), 126.2 (CH, C-1), 125.4 (CH, C-2), 124.7 (C_q, C 9/10), 124.6 (C_q, C 9/10), 124.2 (CH, C-12), 122.1 (CH, C 7), 79.3 (C_q, C 26), 77.4 (CH, C-16), 70.8 (CH_2, C 17-22), 70.7 (CH_2, C 17-22), 70.6 (CH_2, C 17-22), 70.5 (CH_2, C-17-22), 70.1 (CH_2, C 17-22), 70.0 (CH_2, C 17-22), 68.3 (CH_2, C-15), 49.7 (CH_2, C-13), 46.5 (C_q, C-14), 40.2 (CH_2, C-23), 28. 5 (CH_3, C-27) ppm. **FT-IR (ATR):** $\tilde{\nu}$ = 3127 (w), 3039 (w), 2966 (w), 2868 (m), 1704 (vs), 1608 (m), 1504 (s), 1437 (s), 1365 (s), 1275 (m), 1245 (vs), 1170 (vs), 1137 (vs), 1095 (vs), 1040 (vs), 1006 (w), 962 (vw), 879 (vs), 839 (vs), 819 (vs), 759 (m), 727 (s), 706 (vs), 660 (m), 608 (w) cm^{-1}. **HRMS** (ESI,+): found: 1164.5000 $[M]^+$; calc. for $[M]^+$: 1164.5010.

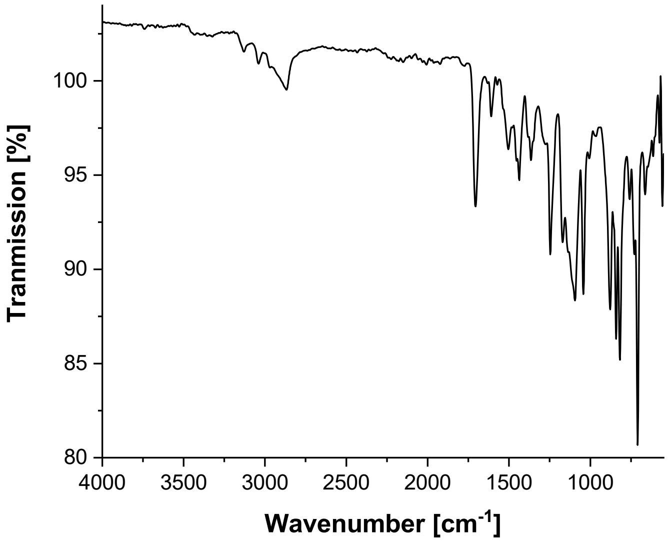

Figure S1. FT-IR ATR spectrum of *tert*-butyl (15-(4-(pyren-2-yl)-1H-1,2,3-triazol-1-yl)-14,14-bis((4-(pyren-2-yl)-1H-1,2,3-triazol-1-yl)methyl)-3,6,9,12-tetraoxapentadecyl)carbamate (**3**).

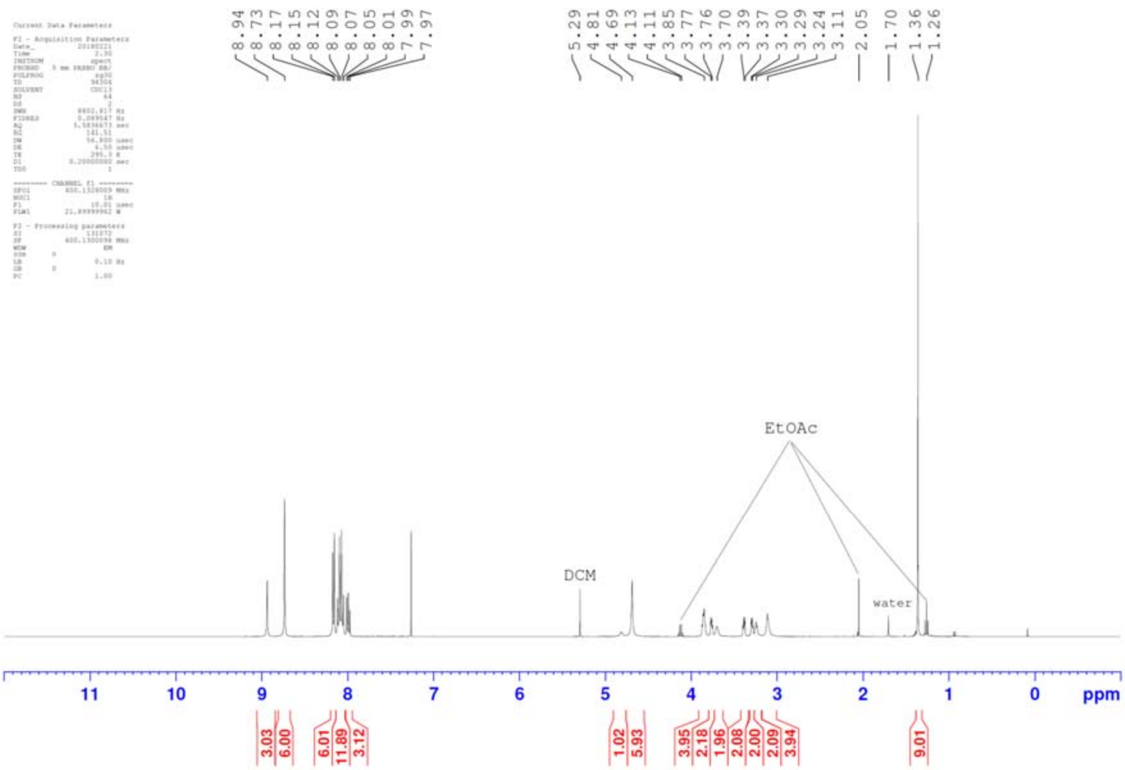

Figure S2. ^{1}H NMR spectrum of (15-(4-(pyren-2-yl)-1H-1,2,3-triazol-1-yl)-14,14-bis((4-(pyren-2-yl)-1H-1,2,3-triazol-1-yl)methyl)-3,6,9,12-tetraoxapentadecyl)carbamate (**3**).

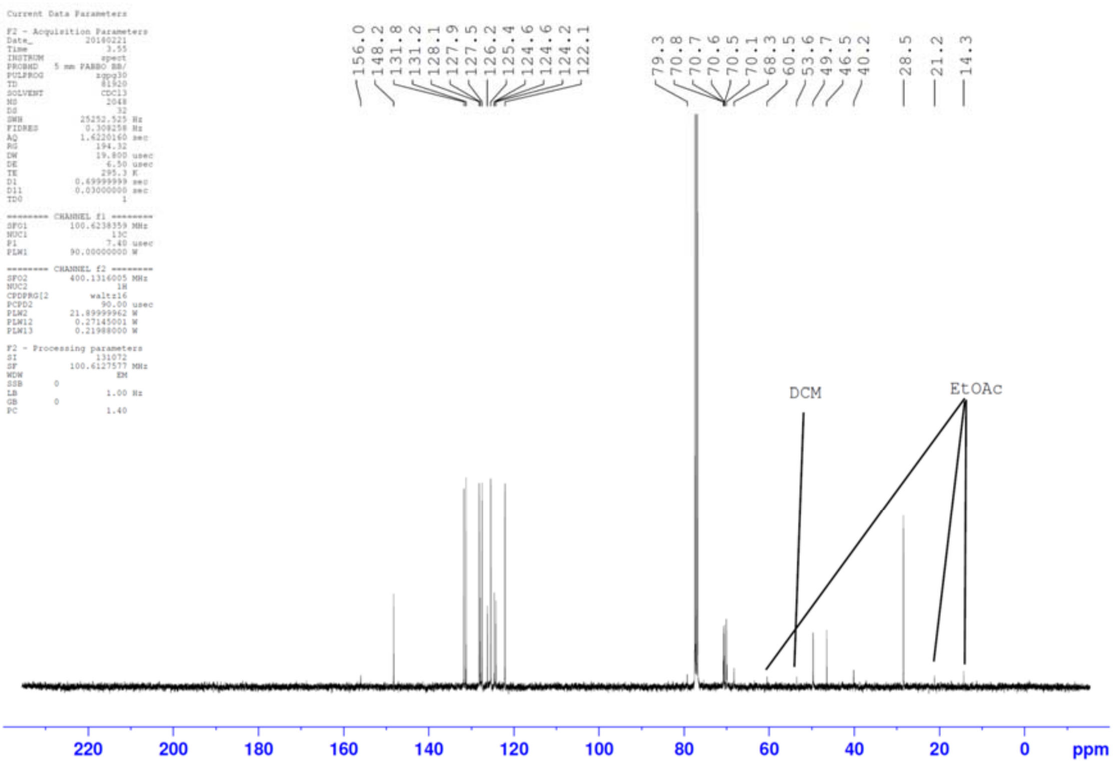

Figure S3. $^{13}C\{^{1}H\}$ NMR spectrum of (15-(4-(pyren-2-yl)-1H-1,2,3-triazol-1-yl)-14,14-bis((4-(pyren-2-yl)-1H-1,2,3-triazol-1-yl)methyl)-3,6,9,12-tetraoxapentadecyl)carbamate (**3**).

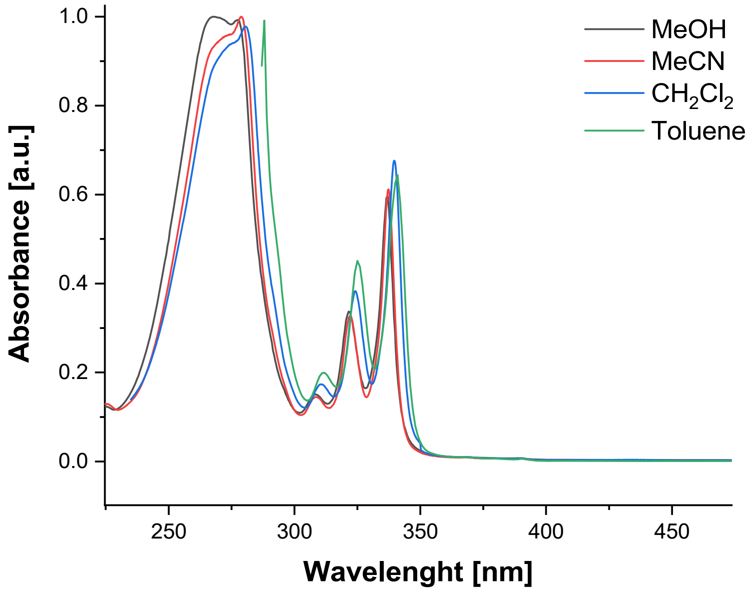

Figure S4. UV/Vis spectra of **3** in methanol, acetonitrile, dichloromethane and toluene (conc. = 4.29 x 10^{-6} mol L^{-1}).

Snapshots of the data obtained from bindfit (http://app.supramolecular.org/bindfit/)

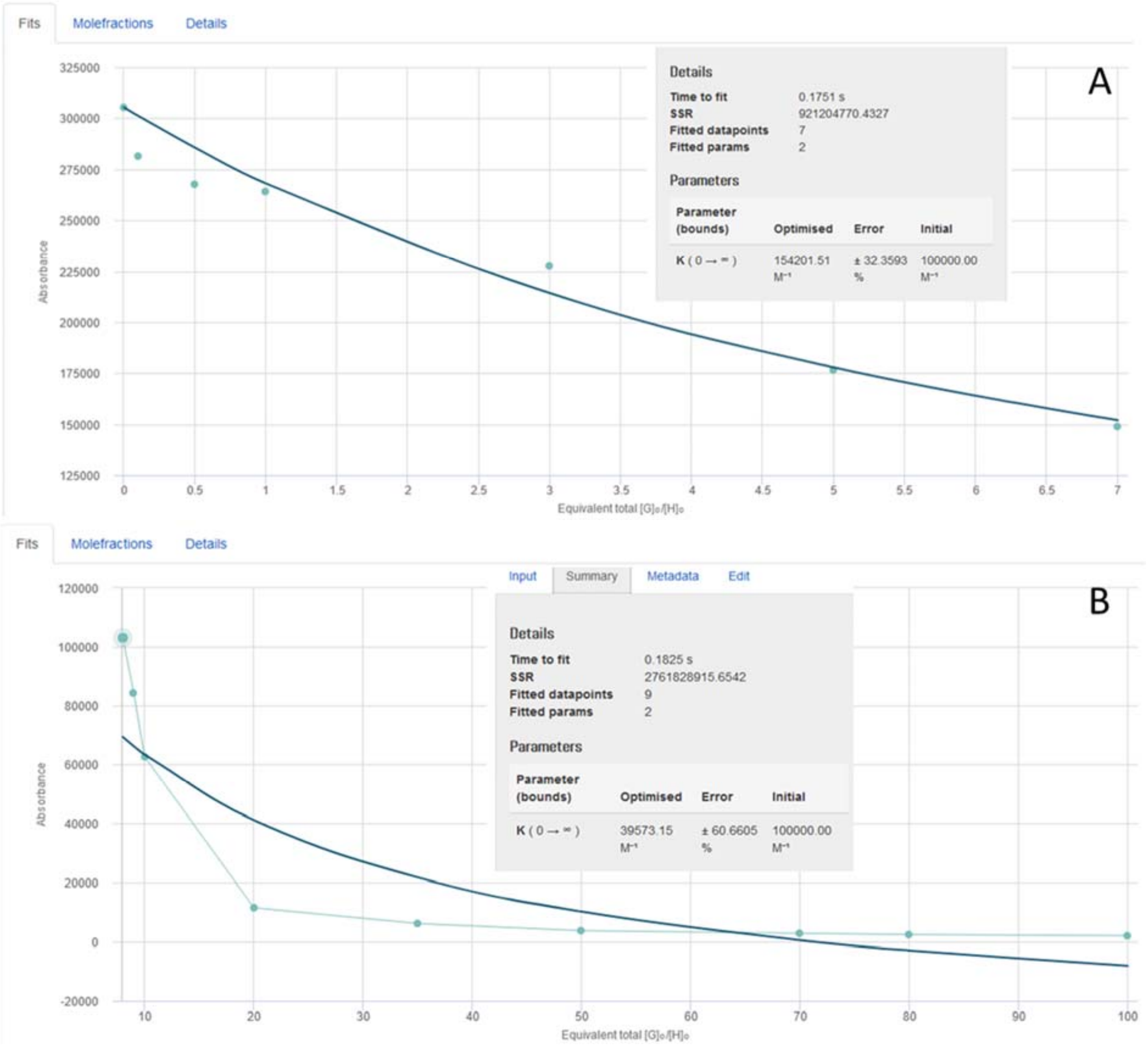

Figure S5. Bindfit plots for Cu^{2+}-titration for A) <10 eq, B) >10 eq using Nelder-Mead fit for 1:1 stoichiometry.

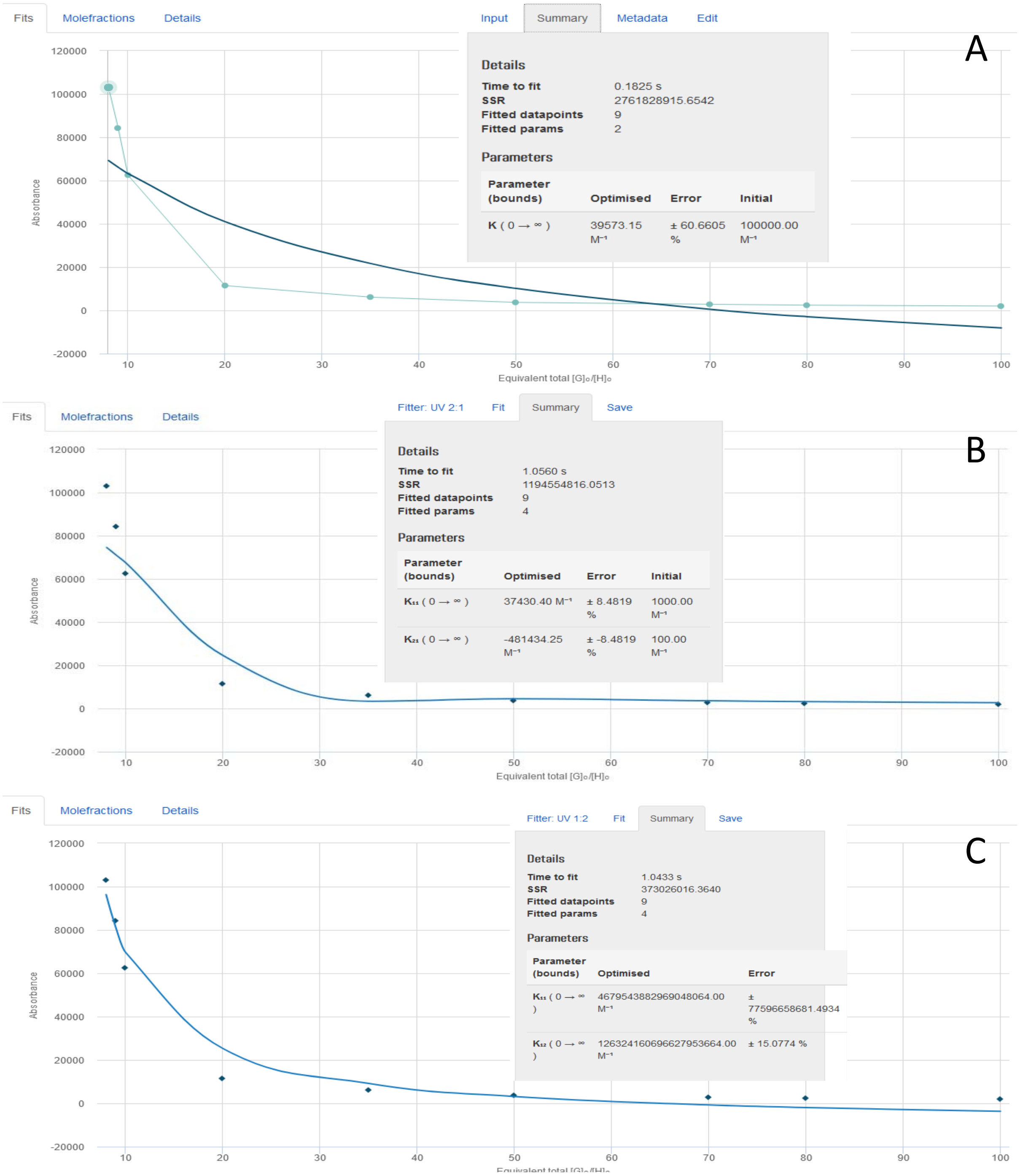

Figure S6. Bindfit plots for Cu^{2+}-titration for >10 eq A) 1:1, B) 2:1 C) 1:2 stoichiometry using Nelder-Mead fit resulting with negative K_{21} for 2:1 and $K_{11} = 4.6 \times 10^8$ M^{-1} for 1:2 stoichiometry.

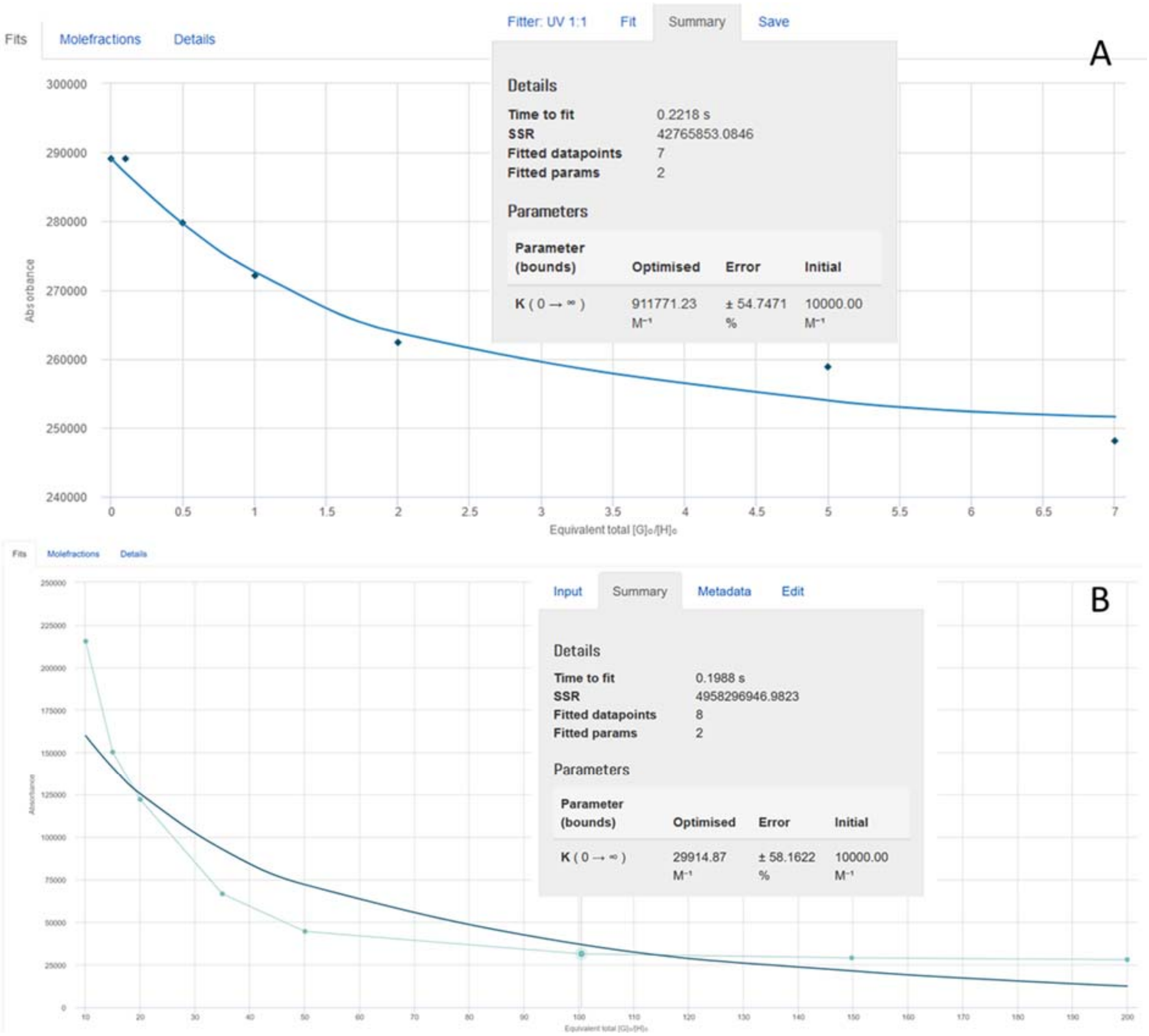

Figure S7. Bindfit plots for Hg^{2+}-titration for A) <10 eq, B) >10 eq using Nelder-Mead fit for 1:1 stoichiometry.

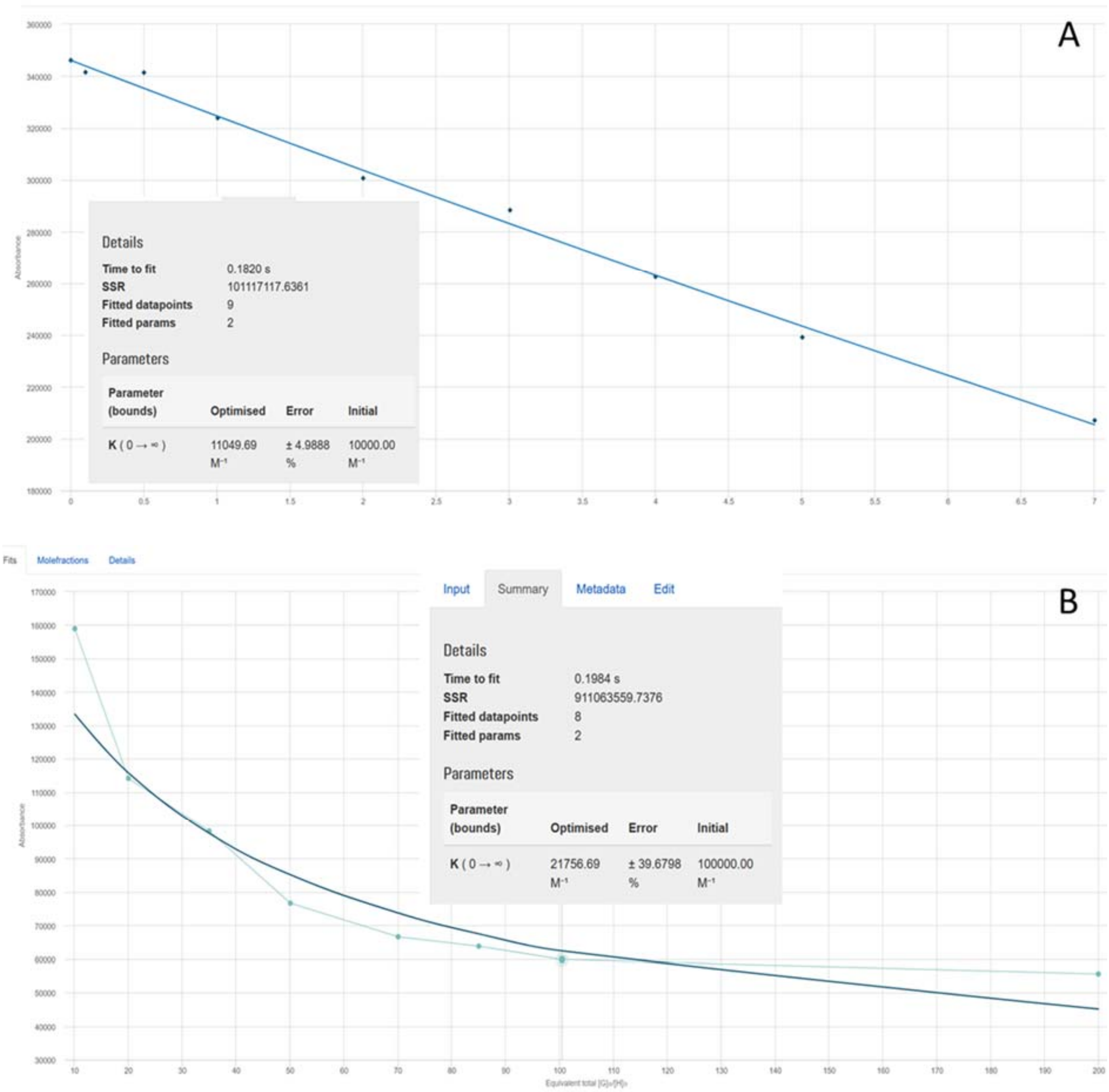

Figure S8. Bindfit plots for Pb^{2+}-titration for A) <10 eq, B) >10 eq using Nelder-Mead fit for 1:1 stoichiometry.

DLS measurements

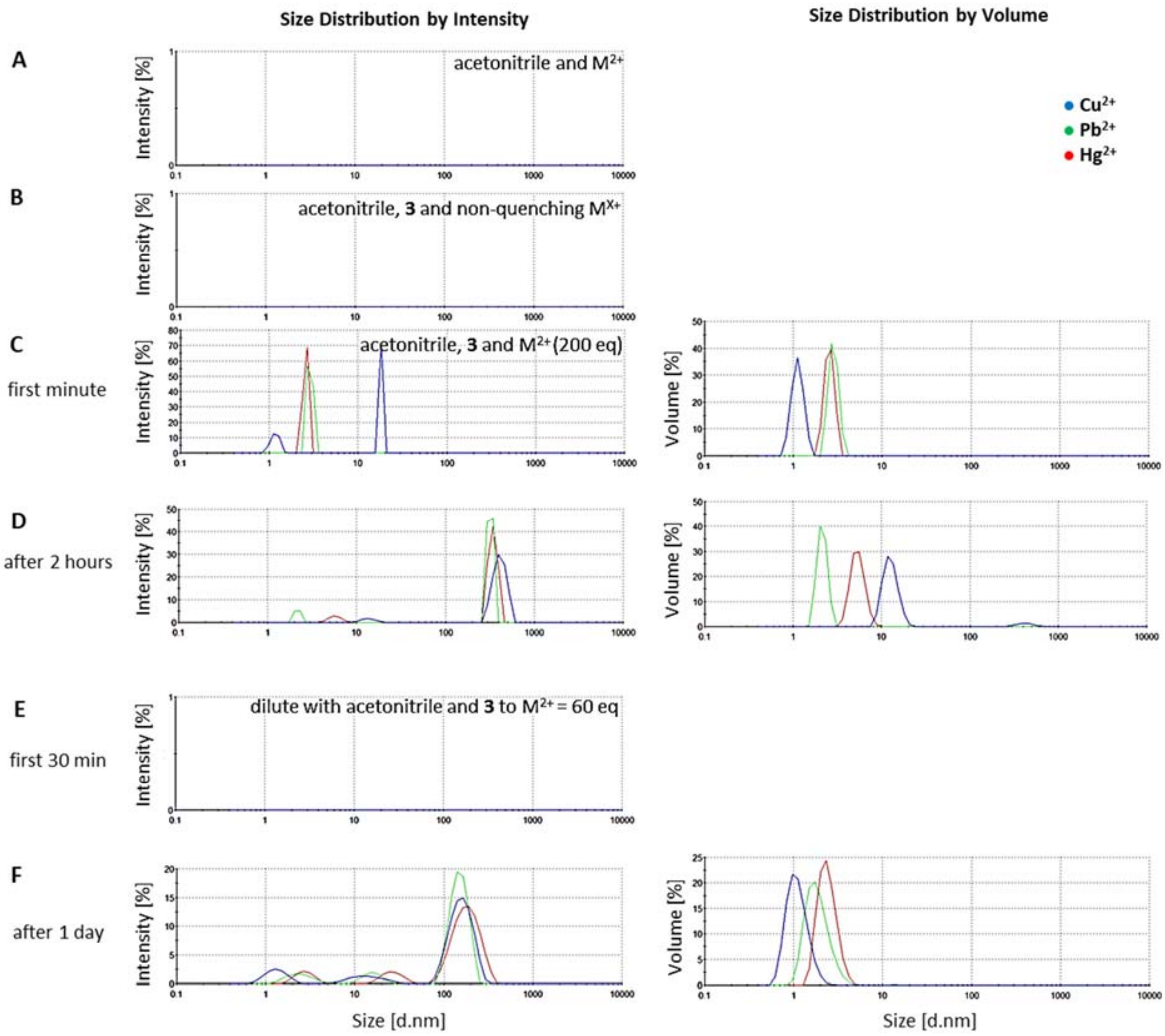

Figure S9. DLS size measurement (diameter) in acetonitrile with A) perchlorate salts, B) chemosensor and non-quenching perchlorate salts, C) first minute of chemosensor **3** (5 x 10^{-6} mol L^{-1}, 1 eq) in presence of 200 eq M^{2+}, D) after 2 hours, E) first 30 minutes of diluted mixture with sensor **3**, acetonitrile and 60 eq M^{2+}, F) after one day.

Benesi-Hildebrand-Plot

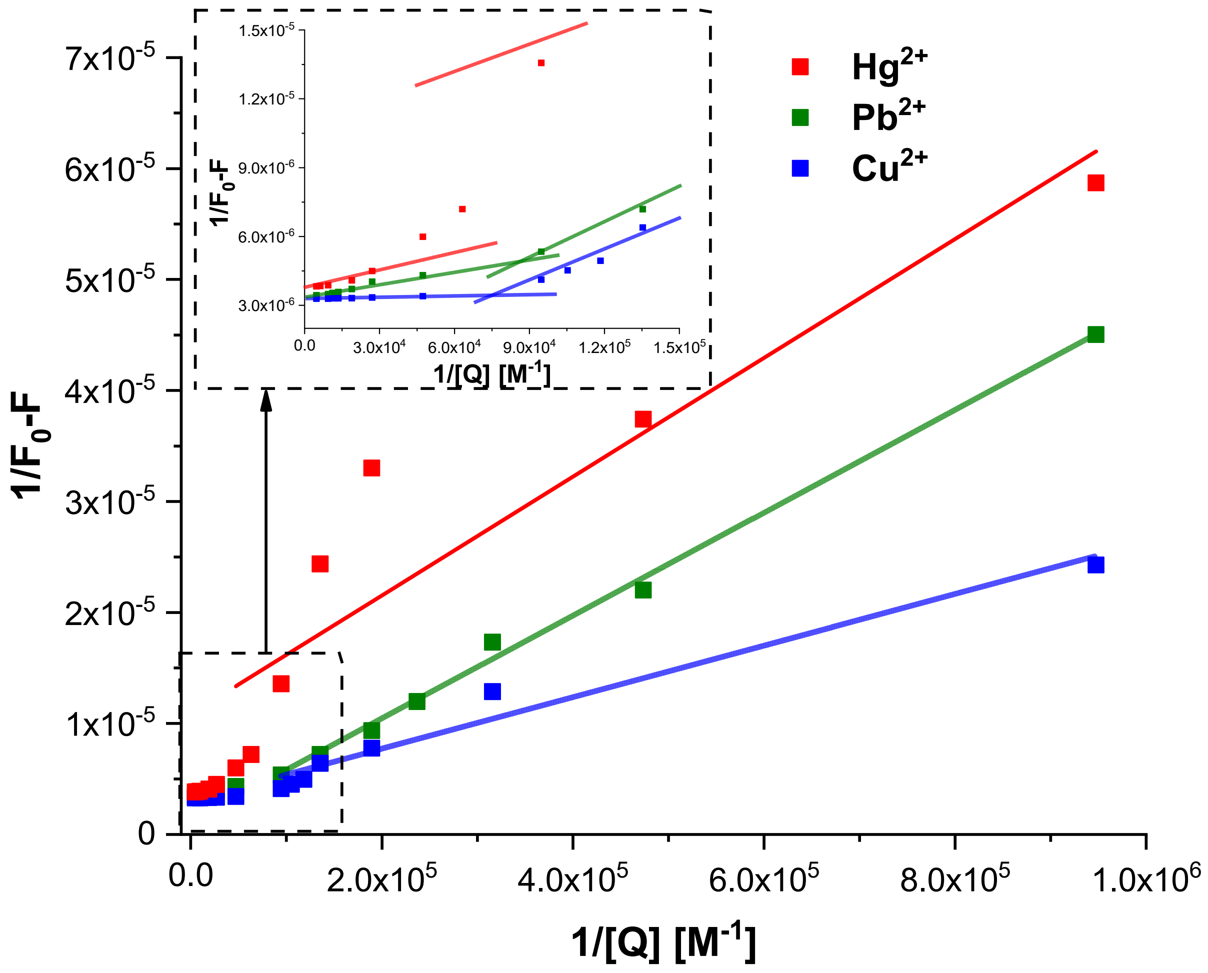

Figure S10. Double reciprocal Benesi-Hildebrand plot of Cu^{2+} (blue), Pb^{2+} (green) and Hg^{2+} (red) titration to **3** with partial regression line for 0-10 eq and 10-200 eq. Inset: zoomed area for 10–200 eq.

The binding constants of the BH-plot and the bindfit methods differ greatly. Because the BH method is only suitable for linear plots, it is not suitable for mixed quenching mechanisms as present here. All linear approximations required for the BH plot ignore the non-linear progression of the values and lead to unsatisfactory results. This is particularly noticeable for the course of the titration with Hg^{2+}, in the range from 0-10 eq, in which the best linear fit does not even comprise any of the experimental values.

^{1}H-NMR-Titration

NMR titration experiments in DMSO-d$_6$ to investigate the ^{1}H-NMR shifts upon addition of the three quenchers.

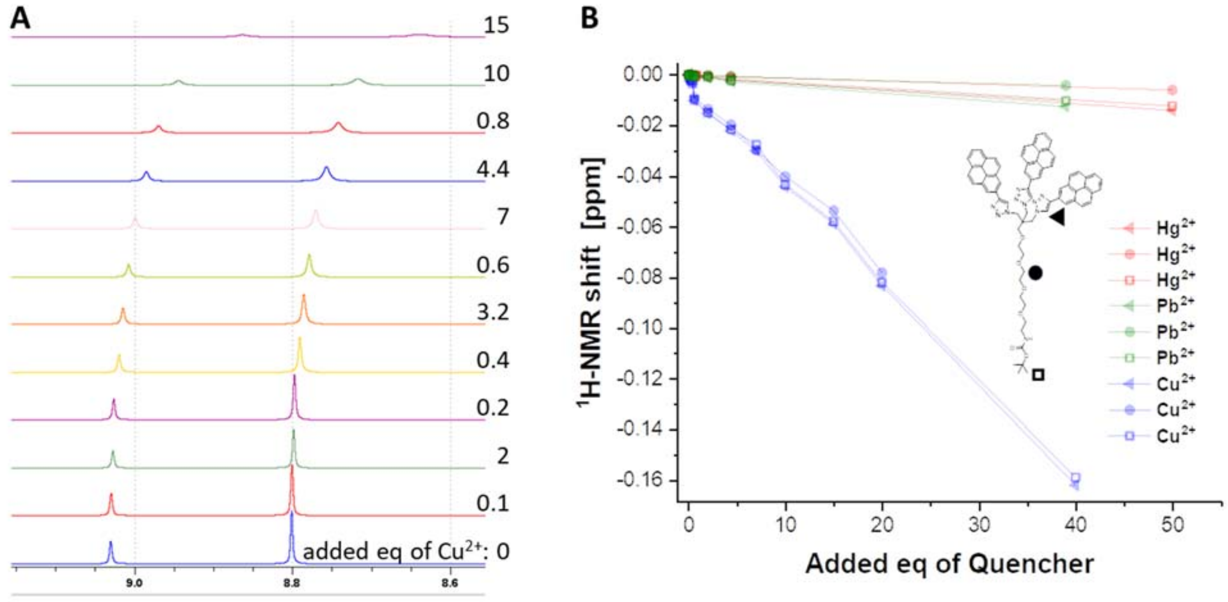

Figure S11. ^{1}H NMR titration results: A) with Cu^{2+} as observed for the aromatic proton signals of pyrene (7-H, 9.2 ppm) and triazole (8.8 ppm), B) the plot of the respective protons shift of the triazole (triangle), the TEG-CH$_2$ (circle) and the CH$_3$ of the Boc group (square) for the respective metal ions.

After the addition of Cu^{2+} (Figure S12, left) the signals not only begin to shift evenly, but also become broader and smaller, which makes it difficult to interpret the spectra from 20 eq Cu^{2+}. Unfortunately, the data show (Figure S12, right) a very linear course without any saturation, which does not allow a calculation of the binding constant. However, the NMR experiments clearly support the results from the fluorescence experiments that Cu^{2+} changes the physical properties of **3** most strongly. It is clearly shown by the small difference in the shift, that the triazole proton (star) is always the most shifted, followed by the protons of the Boc-protective group (square) and the CH$_2$-backbone of the TEG chain (circle). This information suggests that

the interaction between **3** and the metal ion, as shown in Scheme 1, occurs with the whole molecule.

Screening experiments using different non-perchlorate salts

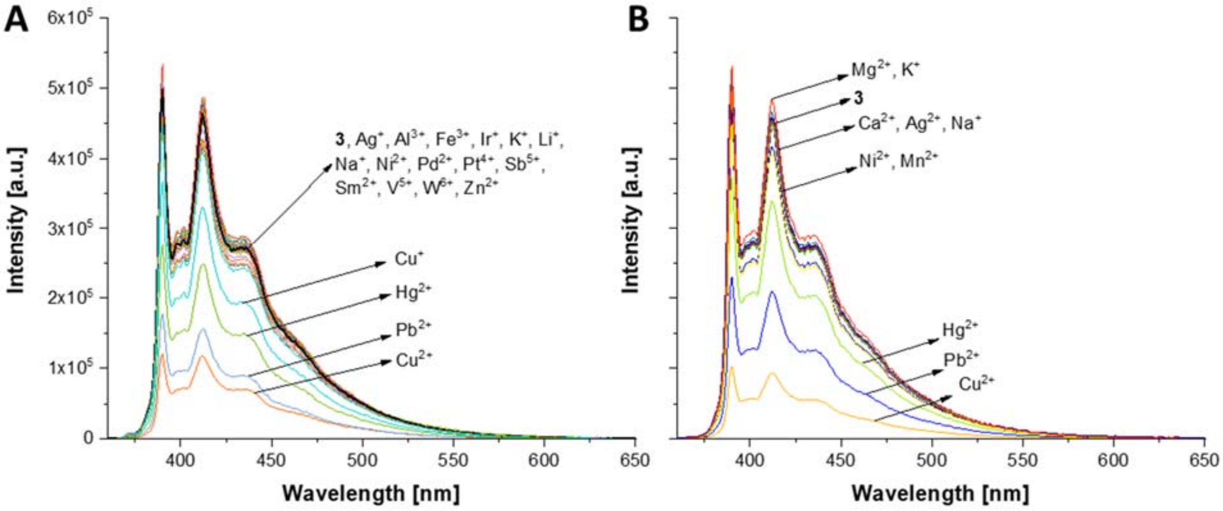

Figure S12. A) Fluorescence spectra in acetonitrile of sensor **3** in the presence of 10 eq of the following salts: $AgNO_3$, $AlCl_3$, $FeCl_3$, $C_{18}H_{30}Ir_2O_2$, CuI, $CuOAc_2$, $HgOAc_2$, KOH, KI, LiOH, LiAc, NaOH, NaI, $NiBr_2$, $PbOAc_2$, $PdAc_2$, $PdCl_2$, $PtCl_6H_2$, $(BrC_6H_4)_3NSbCl_6$, SmI_2, VOF_3, $H_2Na_6O_{40}W_{12}$, $ZnCl_2$ and B) respective perchlorate salts.

Magnification of Figure 2A

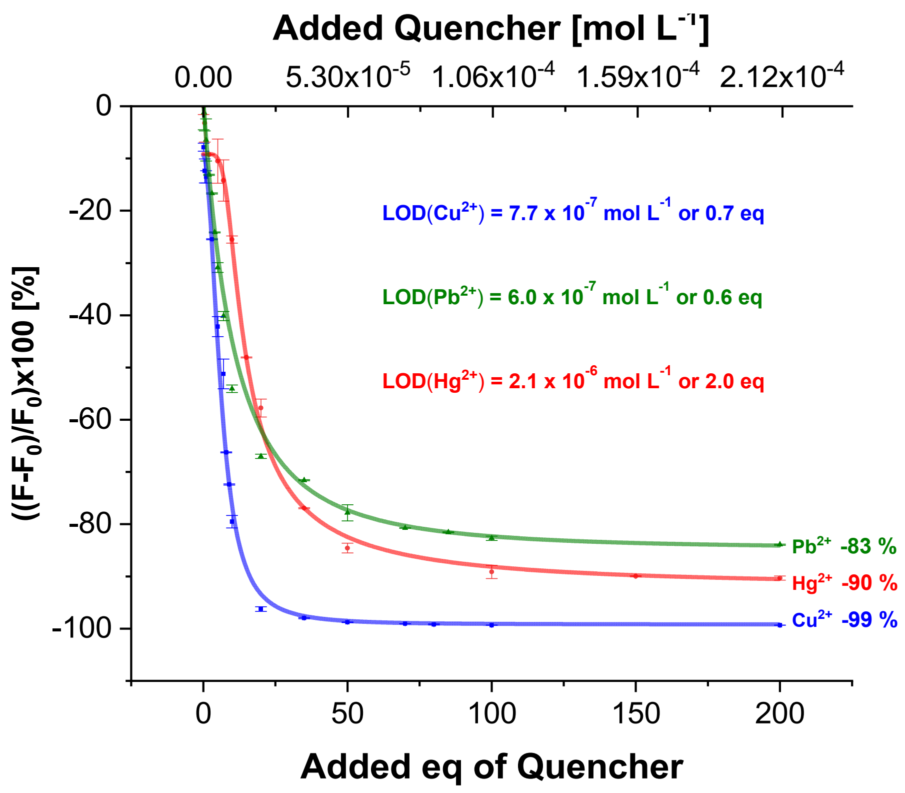

Figure S13. Magnification of Figure 2 A (Fluorescence titration spectra of sensor **3** at 1.02×10^{-6} mol L^{-1} with the quencher ions Cu^{2+}(blue), Pb^{2+} (green) and Hg^{2+} (red), with error bars given for all data points).
